# Fostering Healthy Aging through Evidence-Based Prevention Programs: Perspectives from the Administration for Community Living/Administration on Aging

**DOI:** 10.3389/fpubh.2014.00236

**Published:** 2015-04-27

**Authors:** Michele L. Boutaugh, Laura J. Lawrence

**Affiliations:** ^1^Administration on Aging, Administration for Community Living, U.S. Department of Health and Human Services, Atlanta, GA, USA; ^2^Administration on Aging, Administration for Community Living, U.S. Department of Health and Human Services, Washington, DC, USA

**Keywords:** healthy aging, prevention, evidence-based programs, chronic disease self-management education, older adults, community programs

In 2012, the Administration for Community Living (ACL) emerged as a new operating division within the U.S. Department of Health and Human Services (HHS), bringing together the Administration on Aging (AoA); the Administration on Intellectual and Developmental Disabilities (AIDD), (formerly known as the Administration on Developmental Disabilities); and the Office on Disability. The ACL name reflects both the aspirations of the people we serve and our new mission to maximize the independence, well-being, and health of older adults, people with disabilities across the lifespan, and their families and caregivers (1). Consistent with that mission is a long-standing commitment to the translation of evidence-based prevention programs from the research setting into community practice.

The Administration on Aging continues to administer the Older Americans Act (OAA) (2), which authorizes a national aging network and formula grants to states. These grants fund a wide array of services including congregate and home-delivered meals; transportation; personal and respite care; dementia care; caregiver support services; and programs to protect elder rights (see Figure 1). A portion of OAA funding also supports health prevention and promotion activities. As of 2012, Congressional appropriations require that this funding be used only for evidence-based programs (3).

**Figure 1:**
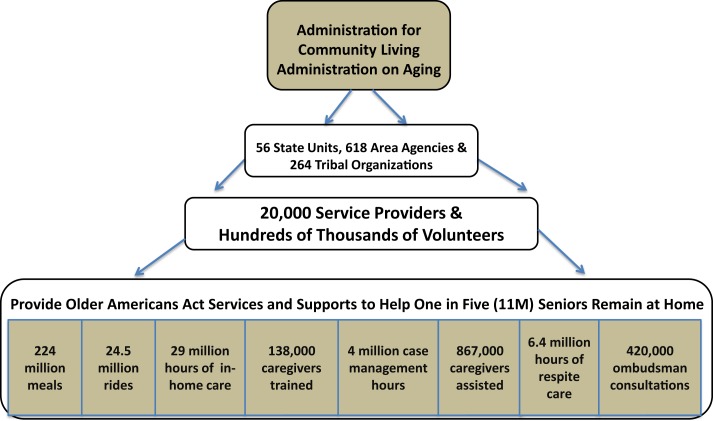
**Aging network structure and Older Americans Act services are shown**. Data from 2012 State Program Reports (SPR) and National Ombudsman Reporting System (NORS). Available from: http://www.agid.acl.gov/DataGlance/

Since 2003, AoA has also provided competitive grants to support collaborations between the aging and public health networks and their partners at the state and local community level. We are especially proud of the impact of these grants, which have helped to forge aging and public health partnerships and to build a program delivery infrastructure in 48 U.S. states and territories. This national infrastructure has enabled over 264,000 individuals throughout the country to participate in evidence-based chronic disease self-management education (CDSME), diabetes self-management training, physical activity, falls prevention, nutrition education, and depression management programs (4).

We are even more excited about what is happening now and the potential that lies ahead. The Patient Protection and Affordable Care Act of 2010 (5) has created many new demonstration projects aimed at achieving the triple aim of healthcare reform: “better health, better health care, and lower costs” (6). These projects provide a tremendous opportunity to demonstrate the value of evidence-based community programs. Programs like the Chronic Disease Self-Management Program (CDSMP) not only improve individual lives, but can be tools to help achieve the “triple aim.” A recent national study of CDSMP, which was partially supported by ACL, demonstrated this potential value. Participants in the study showed significant improvements in health (e.g., self-reported health, pain, fatigue, and depression); experienced measurable improvements in quality of care (e.g., patient–physician communication, medication compliance, and confidence completing medical forms); and required fewer emergency department visits and hospitalizations (7).

These kinds of data have helped our grantees and their partners participate in various health care reform efforts. We are currently supporting 22 grants, all financed by the Affordable Care Act Prevention and Public Health Fund (PPHF). Each of these grants enables state units on aging and state departments of health across the nation to achieve two important goals. They increase the number of older adults and adults with disabilities who complete CDSME programs to maintain or improve their health. They also help agencies build sustainable systems for continuing to deliver such programs after the grant period ends. As a result, many states are utilizing diverse strategies to sustain their programs including embedding programs within other Affordable Care Act initiatives such as patient-centered medical homes and Accountable Care Organizations; partnering with Medicaid and other health insurance providers; collaborating with Federally Qualified Health Centers, Veterans Administration Medical Centers, and other healthcare organizations; and teaming with non-traditional partners such as the Department of Corrections and behavioral health agencies (8).

The PPHF CDSME grants are part of ACL’s larger vision to use partnerships to help reshape healthcare and build a person-centered, comprehensive system that coordinates acute care, long-term care, and community services. For instance, ACL, the Centers for Medicare & Medicaid Services, and the U.S. Department of Veterans Affairs have partnered to invest in a national framework called Aging and Disability Resource Centers (ADRCs). These centers provide a widely accessible “no wrong door” system, where older adults, people with disabilities, and veterans of all ages can learn about and access a full range of long-term care services and supports (9). Many ADRCs are working with hospitals and other partners on care transition programs to better manage discharges from hospital to home or other care settings, and are serving as centralized referral sources for the CDSMP workshops and other evidence-based programs.

We continue to collaborate with other federal and private agencies to address the HHS Strategic Framework on Multiple Chronic Conditions in bringing to scale and enhancing sustainability of evidence-based self-management programs (10). And in September 2014, we released 14 state and tribal falls prevention grants and a new National Falls Prevention Resource Center award financed by the Affordable Care Act PPHF. This new grant program will increase access to evidence-based community programs to reduce falls and falls risk while also increasing the sustainability of such programs through innovative funding arrangements (11).

While proud of what we have achieved, we are also mindful of the challenges that lie ahead. Our goal is to make these programs universally accessible. We have made great progress, but there are still gaps in our coverage. We cannot reach the millions that we still need to reach on our own. We are continuing to work with researchers, foundations, national organizations, and advocacy groups to strengthen our capacity to partner with health care entities and managed care plans. We are also continually exploring effective ways to integrate community-based organizations into new delivery and financing models. ACL is committed to pursuing every opportunity to sustain and expand support for evidence-based prevention programs to improve the lives of older adults and people with disabilities.

## Conflict of Interest Statement

The authors declare that the research was conducted in the absence of any commercial or financial relationships that could be construed as a potential conflict of interest.

This paper is included in the Research Topic, “Evidence-Based Programming for Older Adults.” This Research Topic received partial funding from multiple government and private organizations/agencies; however, the views, findings, and conclusions in these articles are those of the authors and do not necessarily represent the official position of these organizations/agencies. All papers published in the Research Topic received peer review from members of the Frontiers in Public Health (Public Health Education and Promotion section) panel of Review Editors. Because this Research Topic represents work closely associated with a nationwide evidence-based movement in the US, many of the authors and/or Review Editors may have worked together previously in some fashion. Review Editors were purposively selected based on their expertise with evaluation and/or evidence-based programming for older adults. Review Editors were independent of named authors on any given article published in this volume.
